# The Impact of Subpectoral Tissue Expander Applications on Thoracic Deformity: Retrospective Analytic Study on Mastectomy Patients

**DOI:** 10.1007/s00266-025-04850-8

**Published:** 2025-04-29

**Authors:** Mustafa Canberk Gürbüz, Mehmet Akif Cankorur, Ceyhun Uzun, Sevtap Doğan, Atakan Şahiner, Murat Şahin Alagöz

**Affiliations:** 1 Department of Plastic, Reconstructive and Aesthetic Surgery, Kocaeli University Hospital, Eski Istanbul Yolu 10. Km. Umuttepe Yerleskesi, 41000 Kabaoglu, Izmit, Kocaeli Turkey; 2 Department of Radiology, Kocaeli University Hospital, Eski Istanbul Yolu 10. Km. Umuttepe Yerleskesi, 41000 Kabaoglu, Izmit, Kocaeli Turkey

**Keywords:** Breast reconstruction, Tissue expander, Chest wall deformity, Costal deformity

## Abstract

**Introduction:**

With the rising incidence of breast cancer, post-mastectomy breast reconstructions have become increasingly common. Early implant-based reconstructions placed implants subcutaneously, leading to complications, like flap necrosis and implant malposition. Subsequently, the sub-muscular approach gained popularity for its improved outcomes. Tissue expanders have become integral to breast reconstruction, offering a two-stage process with reduced morbidity. However, their use poses challenges, such as chest wall deformities, influenced by a range of variables (age, radiotherapy, and expander volume). This study aimed to explore these correlations.

**Materials and Methods:**

This retrospective study obtained ethical approval and consent was given by 47 patients undergoing immediate two-stage expander-to-implant breast reconstruction between 2013 and 2023. Sterno-costal angles, total expander volume, perioperative filling, and radiotherapy (RT) were evaluated. Pre- and postoperative imaging, including CT and PET-CT scans, assessed chest wall deformities. Patients with osteoporosis, smokers, or pre-existing chest wall deformities were excluded. Standard reconstruction involved creating a sub-muscular pocket, with expanders fixed to the sixth costal periosteum.

**Results:**

Sixty-nine breast of the 47 patients (median age 44 years) were included. Postoperative RT was administered to 46 breasts. Median total expander volume was 360 mL, with an initial fill volume of 45 mL. Postoperative mastectomy wound complications affected eight breasts and were resolved with dressings. Chest wall deformity, indicated by significant postoperative sterno-costal angle changes (*p* < 0.001), was observed in 82.6% of breasts. No significant differences were found between right- and left-sided deformities (*p* = 0.47), nor were correlations noted with RT (*p* = 0.57), total expander volume (*p* = 0.271), or initial filling volume (*p* = 0.759).

**Conclusion:**

This study confirms the association between tissue expanders and chest wall deformities in breast reconstruction. Despite the absence of significant correlations with age, RT, or expander volume, the high incidence of deformities highlights the need for further investigation. Understanding these relationships is crucial for optimizing outcomes in breast reconstruction procedures involving tissue expanders.

**Level of Evidence III:**

This journal requires that authors assign a level of evidence to each article. For a full description of these Evidence-Based Medicine ratings, please refer to the Table of Contents or the online Instructions to Authors www.springer.com/00266.

## Introduction

The escalating incidence of breast cancer has concomitantly increased the frequency of post-mastectomy breast reconstructions. In the early days of reconstruction, implants were situated subcutaneously, positioned beneath the mastectomy flap and above the pectoralis muscle. Although this method proved straightforward and maintained muscular integrity, its deficiency in overlying tissue support engendered numerous complications, including flap necrosis, capsular contracture, and implant malposition (“bottoming out”) [1]. Since then, the sub-muscular approach, wherein the implant is positioned beneath the pectoralis and serratus anterior muscles, has superseded subcutaneous reconstruction [2].

The use of tissue expanders has become widespread in breast reconstruction following mastectomy. Two-stage breast reconstruction, employing a tissue expander followed by a breast prosthesis, is regarded as a straightforward procedure with reduced donor site morbidity and abbreviated surgery duration. While the muscular pocket provides adequate tissue coverage and confines implant mobility, it is concurrently associated with complications, such as hematoma, animation deformity, loss of muscular function, and chronic pain [3–5]. Moreover, muscle contraction against the tissue expander (TE) can induce deformation of the chest wall [6, 7].

Various factors impact the outcomes of two-stage breast reconstruction, including smoking, obesity, and the chosen reconstructive method, all of which have been identified as correlates with the risk of complications in implant-based reconstruction [8, 9]. Another influential determinant of the surgical outcomes in two-stage breast reconstruction is the expansion protocol. Despite the endorsement of reconstructive surgery following, it is notable that initial saline administration immediately following expander insertion has been shown to augment its final volume by up to 30%, although alternative studies have reported complete deflation [10]. These commonplace complications have been thoroughly elucidated in the extant literature.

We posit that a conceivable correlation exists between the use of TEs and the temporary or permanent alteration of the chest wall. Other reliable techniques have been shown in similar studies in the literature to quantitatively demonstrate chest wall deformity. We wanted to develop sterno-costal angle measurements as an alternative to these techniques [11]. In this context, we present a study predicated on computed tomography (CT) scans of the chest wall subsequent to the application of TEs.

### Patients and Methods

**Fig. 1:**
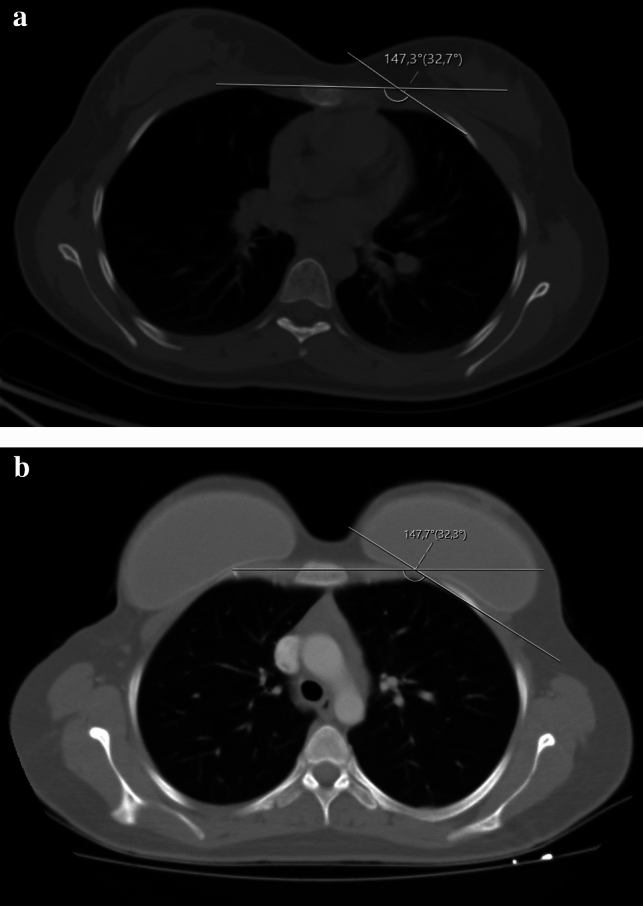
Preoperative (**a**) and postoperative (**b**) evaluations depict a patient who underwent bilateral two-stage expander surgery. There is an absence of any observed costal deformity between the two stages of surgical intervention

The study received approval from the institutional ethical committee, and informed consent was obtained from all patients. We conducted a retrospective investigation focusing on the sterno-costal angles, total amount of TE volume, peroperative TE filling, radiotherapy (RT) treatment of individuals who underwent immediate two-stage expander-to-implant reconstruction between 2013 and 2023 in Kocaeli University Hospital. The primary objective was to assess the occurrence of chest wall deformities. In the study, we evaluated the preoperative healthy costas of the patients as the control group and the costas of the same patients who were exposed to expanders as the study group. We thought that this evaluation would give us the best reference. Standard preoperative and postoperative oncologic imaging, including positron emission tomography-CT (PET-CT) and CT scans, was used. For bilateral mastectomy patients, each breast was evaluated independently (Fig. 1). Patients with a prior history of osteoporosis, were active smokers, or a history of chest wall deformity or malformation were excluded from the study. All analyzed patients underwent a two-stage reconstruction following mastectomy. The initial surgery occurred immediately after mastectomy, involving the creation of a complete sub-muscular pocket beneath the pectoral major muscle. The inferior part of the expander was fixed to sixth rib costal periosteum. All patients received the same type of expanders. In our practice, we use Mentor Siltex Contour Profile Breast Tissue Expander with integral injection dome 350 or 450 cc volume dependent on patient’s breast footprint. The expansion started 14 days after the mastectomy or 14 days after the last RT and was performed at 7–10-day intervals.

### Imaging System

**Fig. 2:**
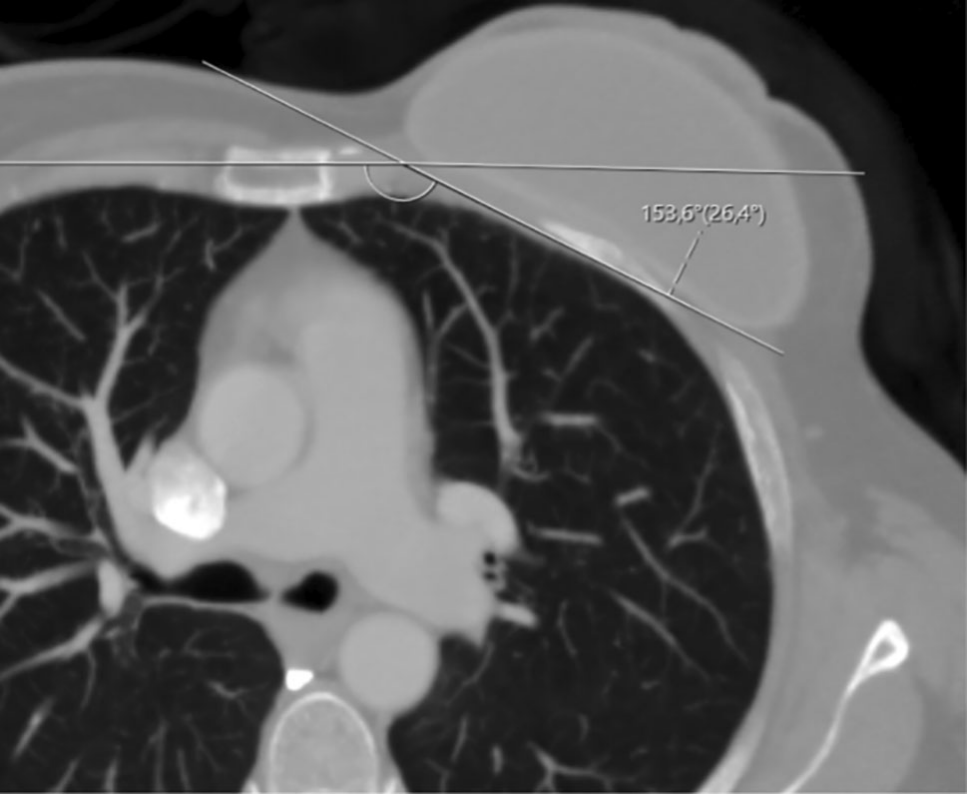
Angles were assessed within axial planes, and a line running parallel to the ribs was traced from the inner surfaces of the third, fourth and fifth ribs. Another line was drawn parallel to the plane of the sternal manubrium at the corresponding level for each rib. The angle formed by the intersection of the sternal and costal lines was noted

Preoperative (before mastectomy operation) and postoperative (after second-stage expander operation) cross-sectional images of the patients included in the study were evaluated, and angle measurements were assessed. Measurements were made using CT if possible, or PET-CT if CT was not available. Measurements were performed by two radiologists (SD and MAC) with 15 years and 3 years of experience in thoracic radiology. One of the radiologists studied the preoperative and the other postoperative images and evaluated the angle measurements independently. Angles were measured in axial planes, and a line parallel to the rib was drawn from the inner surfaces of the third, fourth, and fifth ribs. A second line was drawn parallel to the sternum manubrium plane at the same level. The intersection angle of the sternal and costal lines was recorded (Fig. 2). The average of the angles obtained for all three ribs was taken and included in the study as the final angle value.

### Statistical Analyses

Statistical evaluation was performed with IBM SPSS, version 29.0 (IBM Corp., Armonk, NY, USA). Compliance with normal distribution was examined with Shapiro–Wilk and Kolmogorov–Smirnov tests. Variables with normal distribution are given as mean±standard deviation (SD), and variables with non-normal distribution are given as median and interquartile range (IQR). The difference between groups was investigated by independent samples t test. Paired samples t test was used for dependent group comparisons. Relationships between numerical variables were determined by Spearman correlation analysis. In hypothesis tests, a *p* <0.05 was considered sufficient for statistical significance.

## Results

In this study, 362 patient underwent mastectomy and expander surgery between December 2013 and January 2023. Of these, 47 (13%) met the inclusion criteria and 21 (44.7%) patients had bilateral and 26 unilateral breast cancer; thus, a total of 69 breasts were included in study. Out of the 69 breasts, 33 (47.8%) were right sided. All of the patients underwent total sub-muscular pocket (pectoral major, serratus pocket) reconstruction with an immediate TE.

The patients had a median (IQR) age of 44 (37–50) years. Forty-six (66.7%) breasts received postoperative RT between the two stages of reconstructive surgery. Overall, the median TE volume was 360 (330–427.5) mL. The median initial TE fill volume was 45 (30–100) mL. Eight breasts exhibited postoperative mastectomy wound healing issues, including blistering, and this complication was resolved with dressings. There were no patients in this series who experienced major post-surgical infections or required additional surgery.

**Fig. 3:**
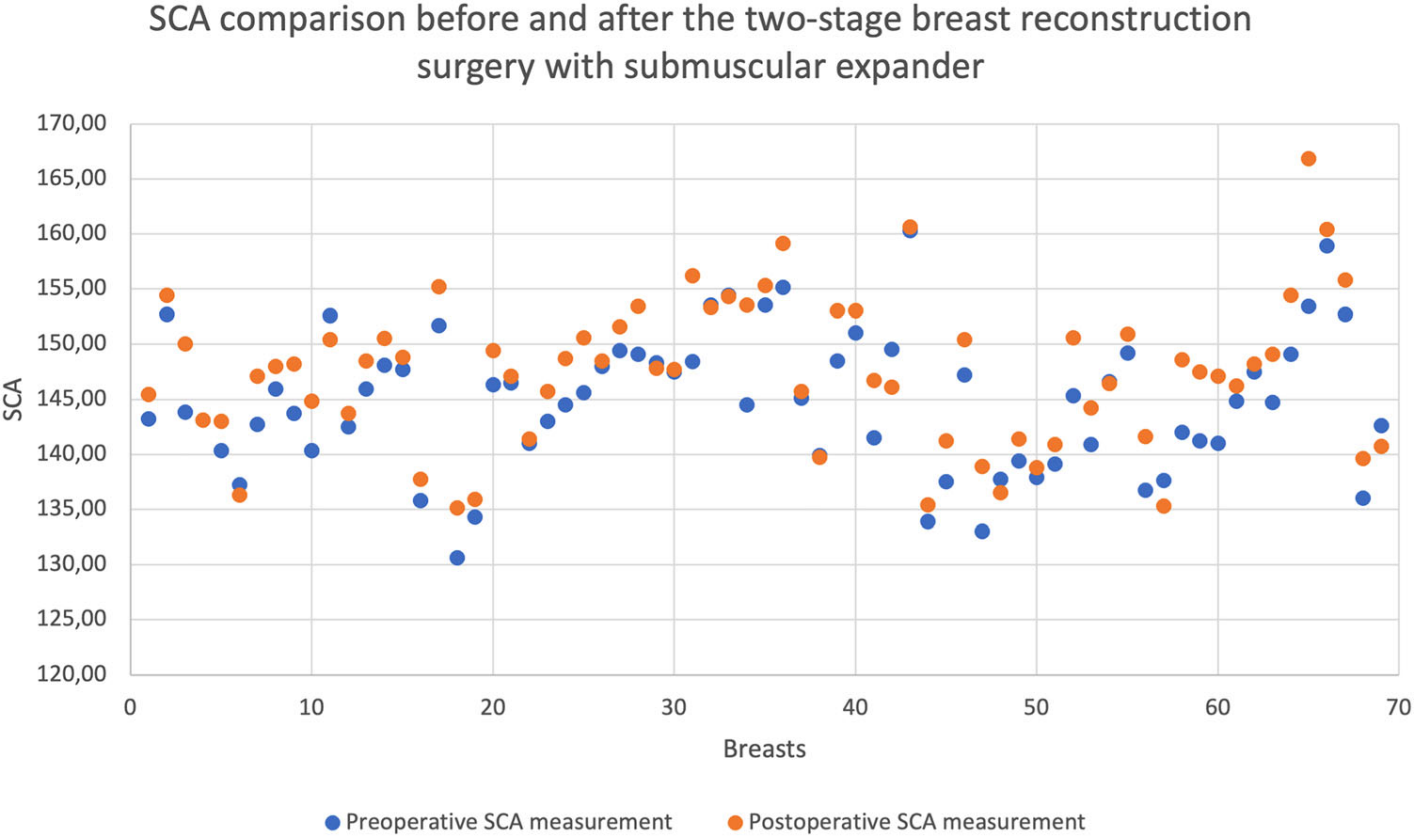
SCA comparison before and after two-stage breast expander surgery. Preoperative and postoperative patient evaluation. Graphic demonstrates angle differences for each patient

The expansion started 14 days after the mastectomy or 14 days after the last RT. Expansion procedures were performed at 7–10-day intervals with a median of 46 (40–50) mL fills at each visit. The mean±SD preoperative sterno-costal angle (SCA) was 144.8 ± 6.2°, and the mean postoperative SCA was 147.4 ± 6.6°. Comparing the preoperative and postoperative SCAs, there was significant change (*p* < 0.001) between costal angles (Fig. 3). There was no significant difference between right- and left-sided costal deformity (*p* = 0.47). There was no correlation between RT and costal deformity (*r* = 0.97, *p* = 0.57); neither were there correlations between total TE filling volume (*r* = 0.13, *p* = 0.271) nor initial peroperative expander filling volume (*r* = 0.038, *p* = 0.759). Age was not related to change in SCA (*r* = 0.1, *p* = 0.39) (Table 1).

**Table 1:** Correlation between the disparity in costal angles and variables such as total expander volume, perioperative inflation quantity, and patient age was assessed through the determination of correlation coefficients

	Costal angle percentage change	Total TE volume	Peroperative TE filling volume	Age
Median	2.21	360	45	44
Percentiles (25-75)	0.64–4.4	330–427	30–100	37–50
*p* value	–	0.27	0.75	0.39
Correlation coefficient	1	0.13	0.038	0.10

## Discussion

**Fig. 4:**
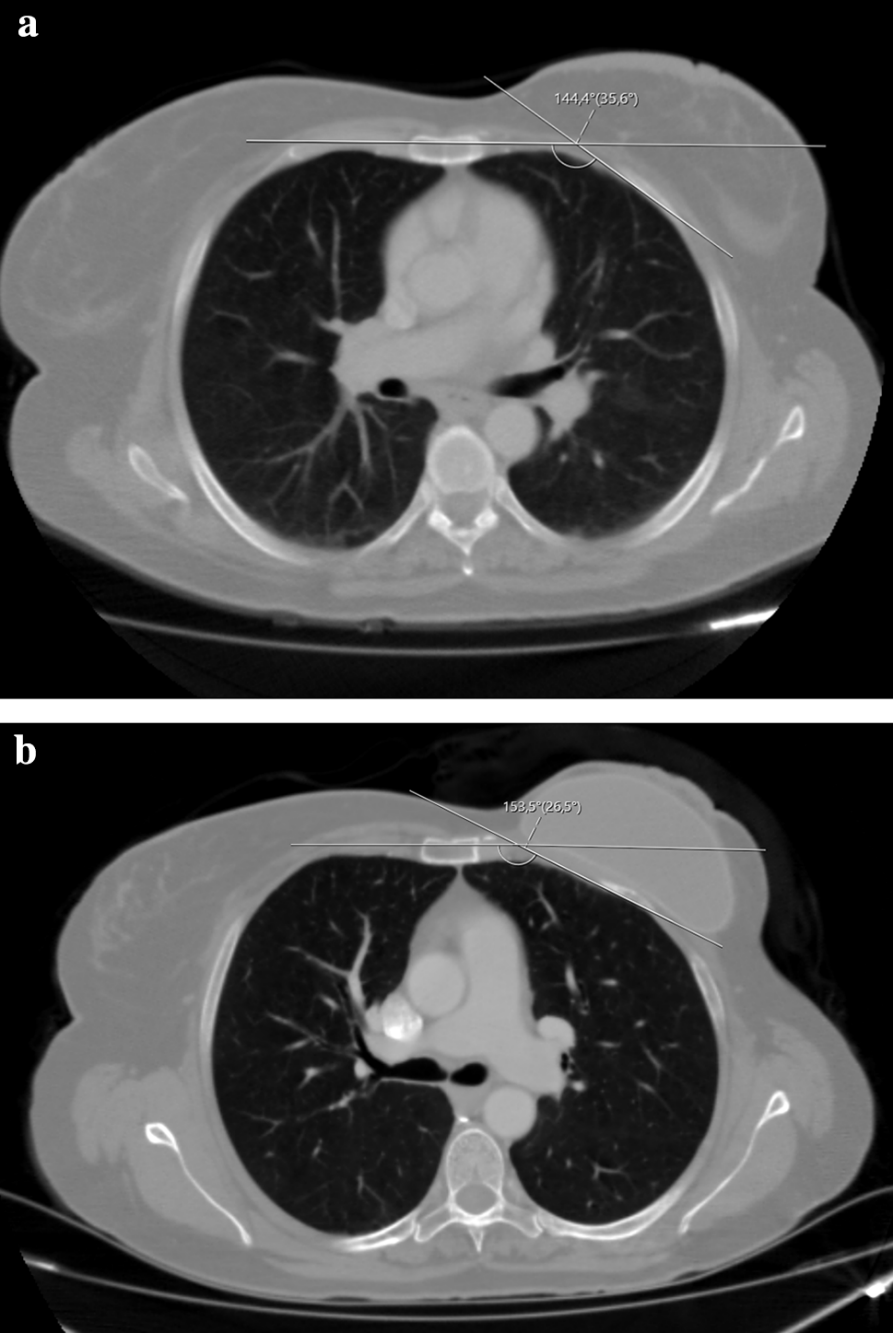
Picture **a** : PET-CT imaging reveals the anatomical condition of a patient during the preoperative evaluation preceding the initial stage of expander surgery. Picture **b**: taken subsequent to the second-stage expander surgery with a permanent implant, a notable observation is a 9.1° flattening of the fourth rib compared to its appearance between the two stages of surgical intervention

**Fig. 5:**
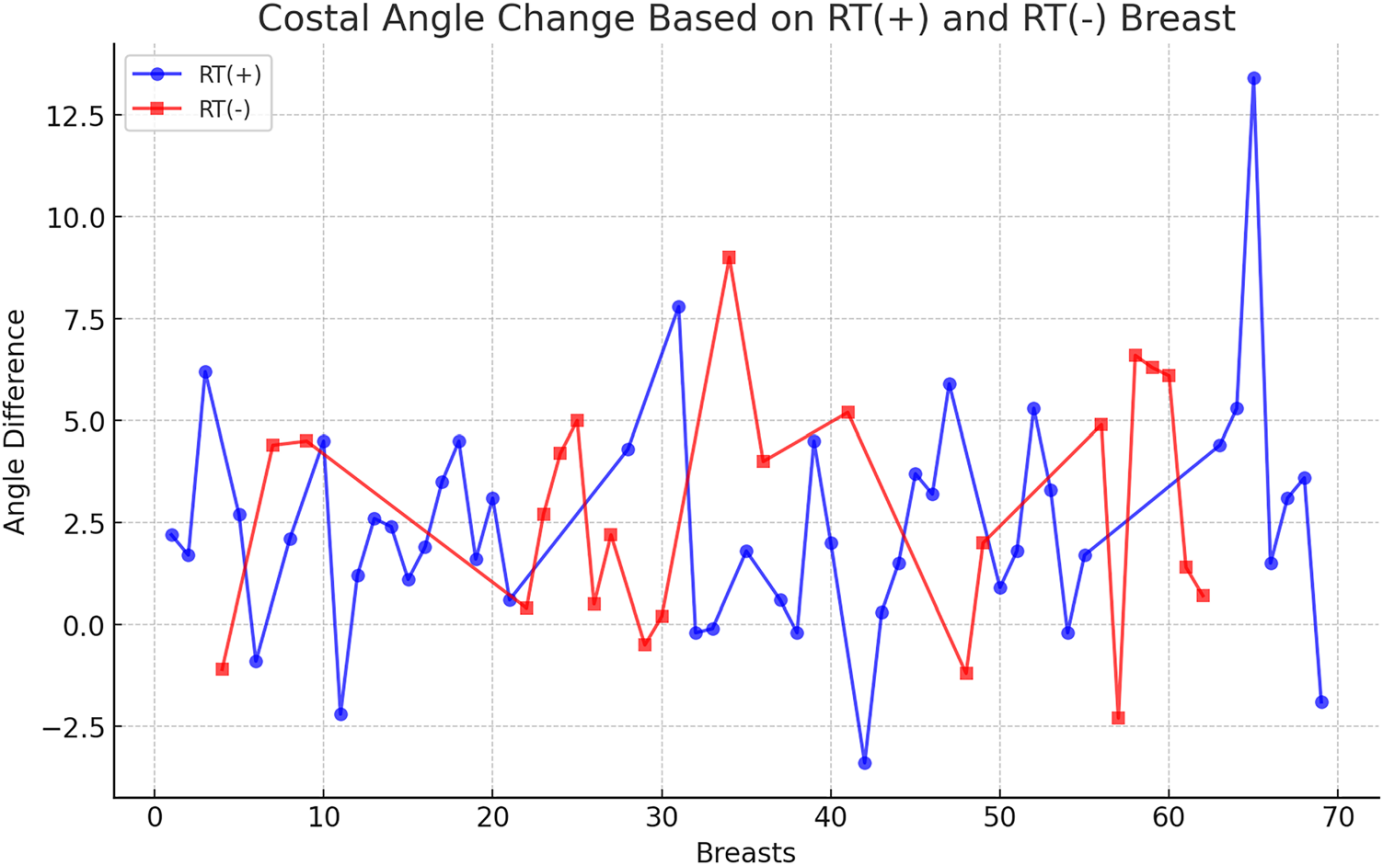
Costal angle change based on RT (+) and RT(−) Breasts. The table shows the SCA changes in breasts that received radiotherapy(46) and breasts that did not(23). As seen in the table, there is no significant difference between the two groups

**Fig. 6:**
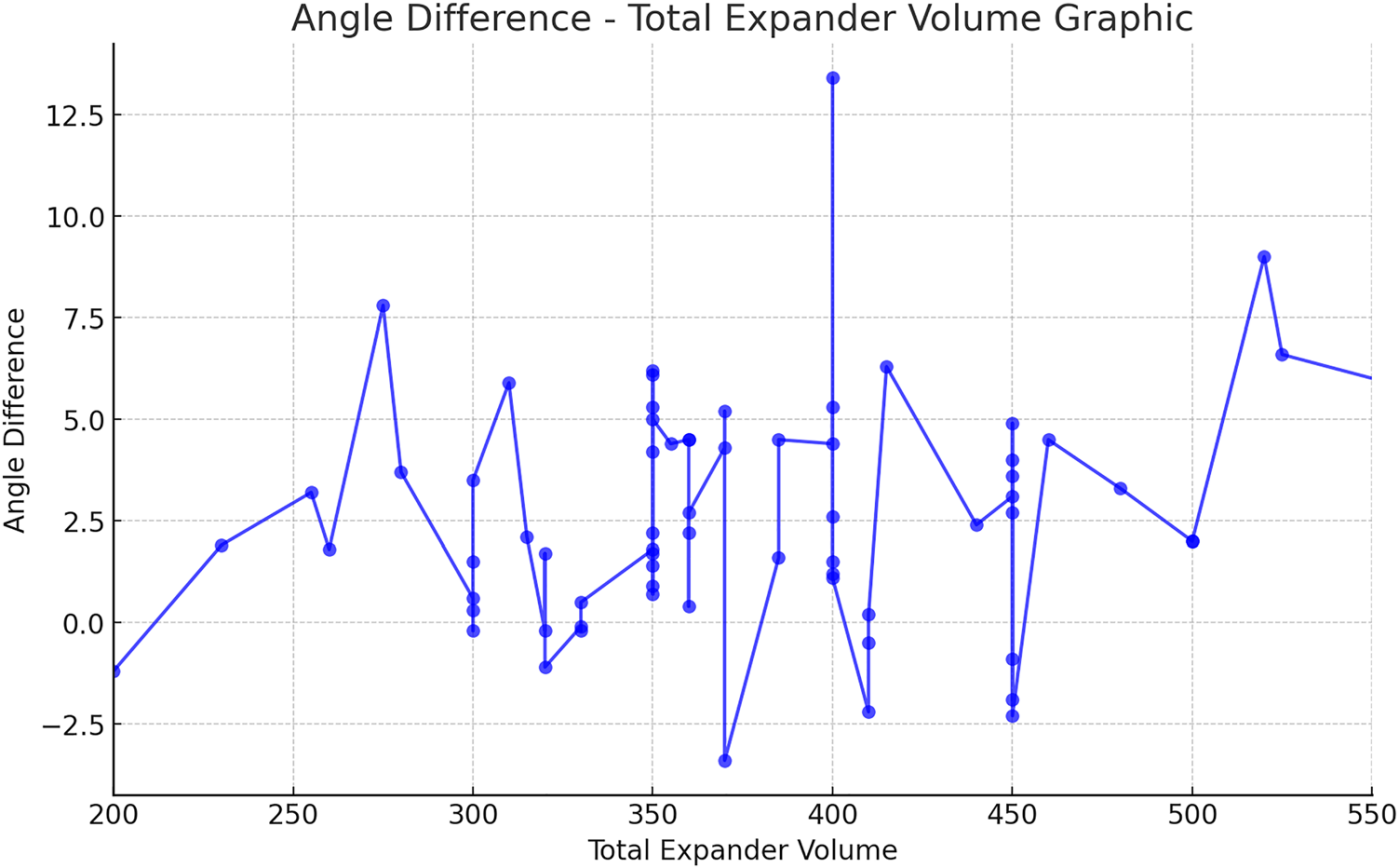
Costal angle difference—total expander volume graphic. Graph showing the angle change of SCA with the total expander inflation amount. Median angle change was 1.75 in 30 expanders inflated to ≤ 350 cc; the median angle change was 2.7 in 39 expanders inflated to > 350 cc. Although this shows the increasing angle at high inflation amounts, it does not give a statistically significant value

**Fig. 7:**
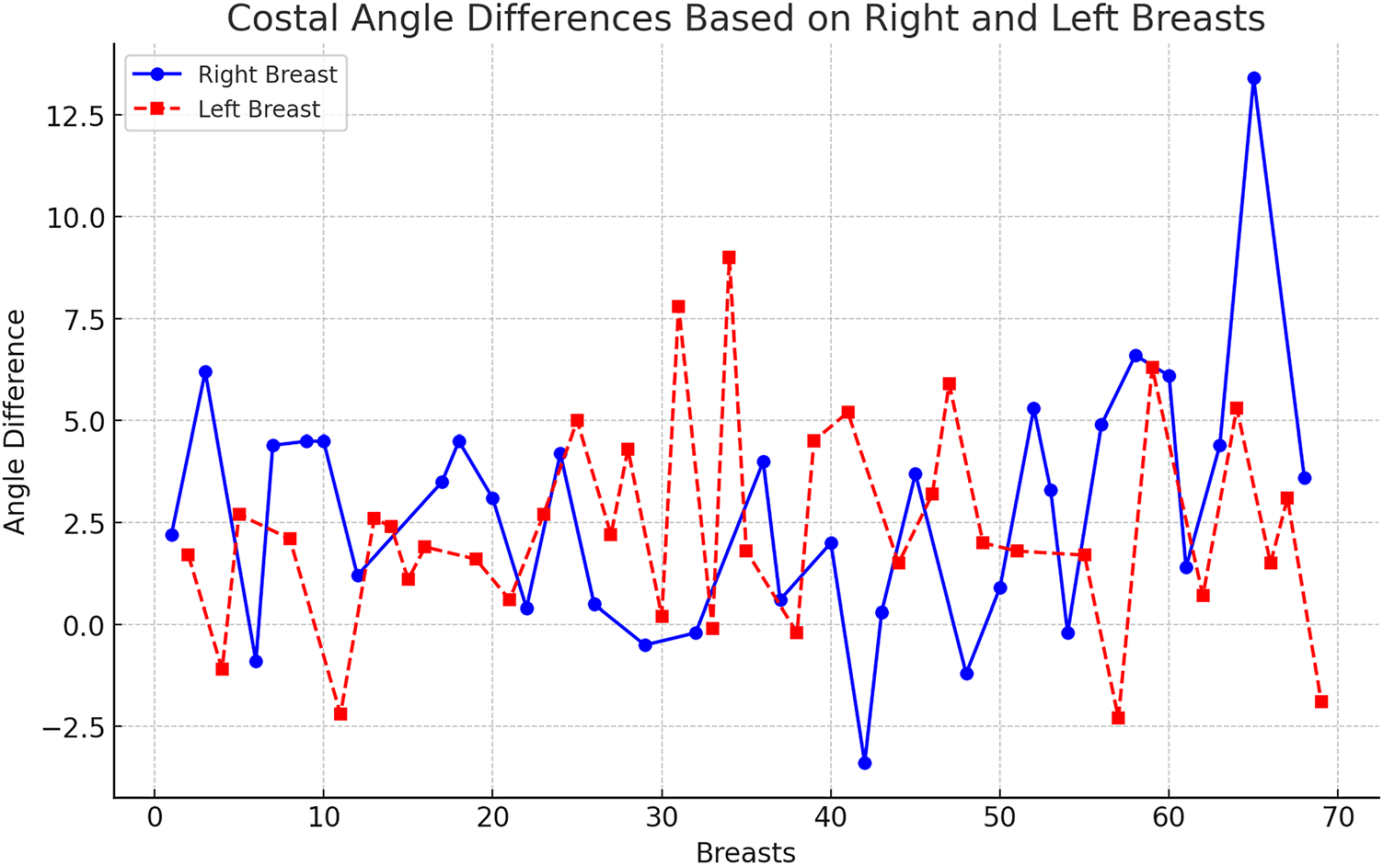
Costal angle differences based on right and left breasts. In the table, you can see the comparison of the SCA change of the expander cases applied submuscularly in the right (33) and left (36) breasts

**Fig. 8:**
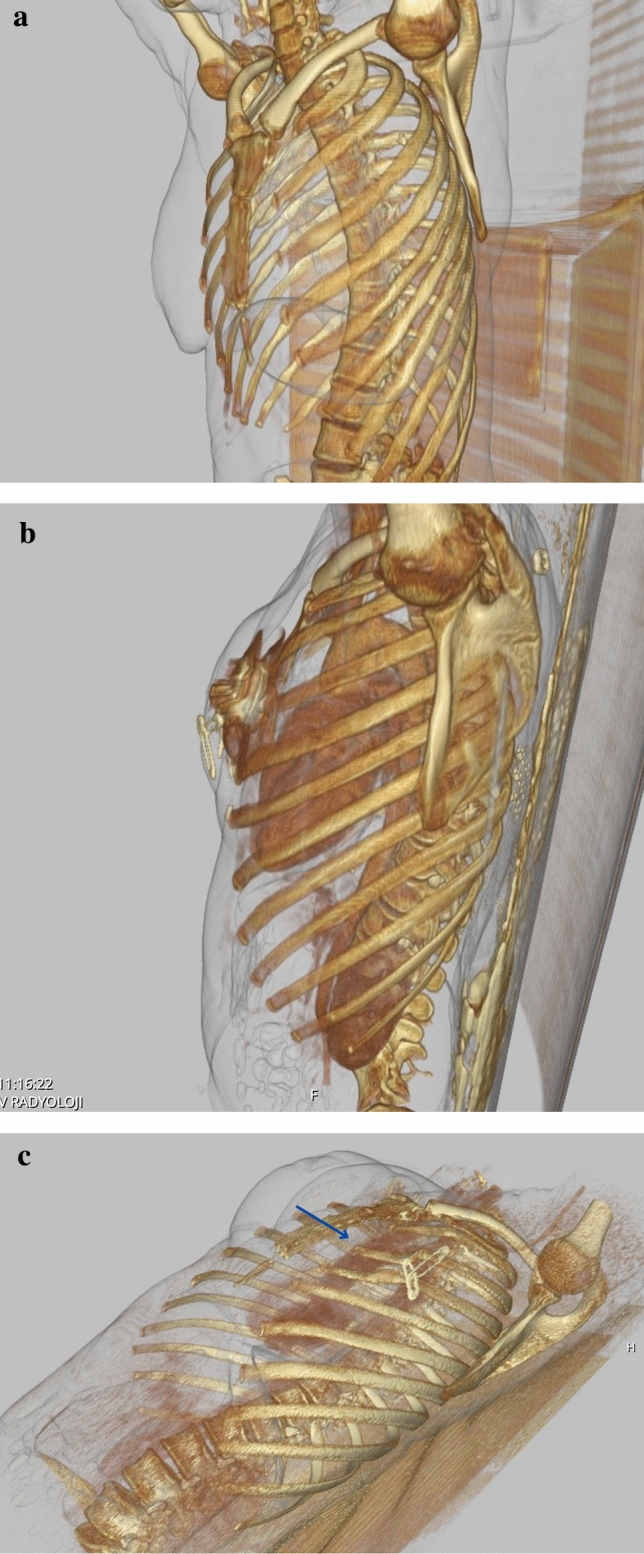
In the images above, you can see the 3D thorax CT of the same patient in the preoperative (**a**), early postoperative expander 1st-stage (**b**), and early postoperative expander 2nd-stage (**c**) periods. The remodeling in the 3rd rib corresponding to the center of the expander is clearly visible (blue arrow)

**Fig. 9:**
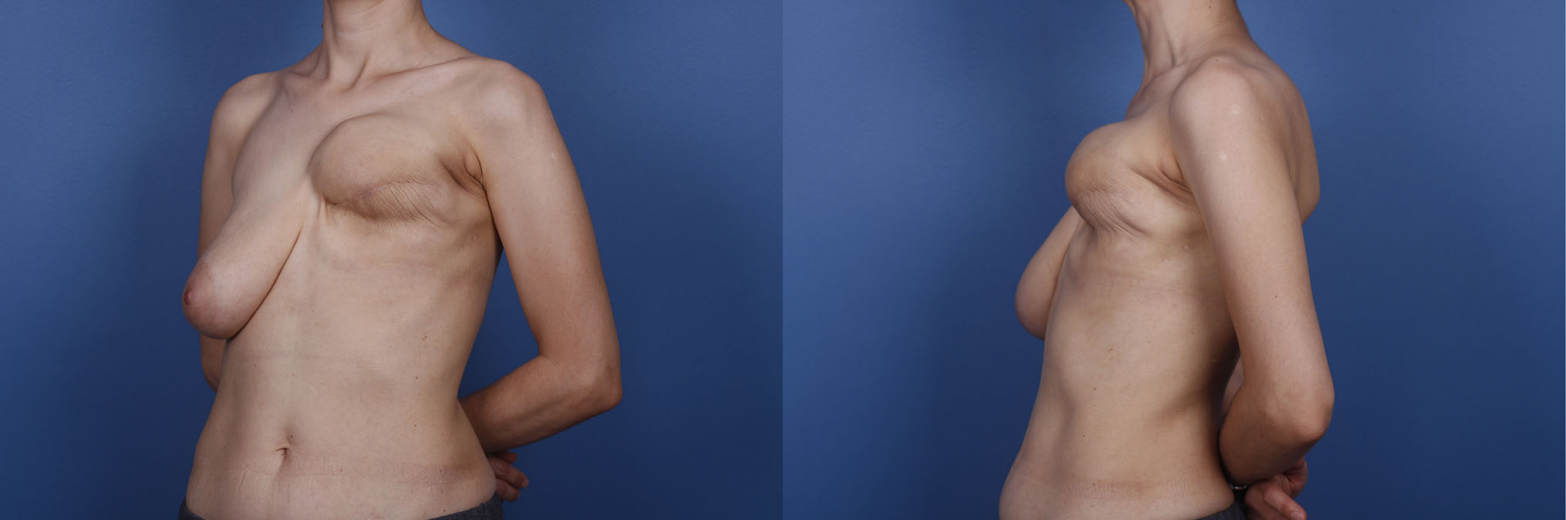
Photograph of the same patient before the expander 2nd session operation. You can see capsular contracture and deformed skin due to radiotherapy treatment

The findings of this investigation confirm that the use of tissue expanders in breast reconstruction procedures may induce a deformity in the chest wall. In the present study, 82.6% (57/69) breast exhibited a chest wall deformity with the expander (Fig. 4). The precise factors contributing to such deformities remain elusive. Our inquiry sought to ascertain whether age might correlate with the incidence of chest wall deformities, yet we found no significant association, albeit there was an absence of elderly patients in our cohort, with the oldest being 62 years. Notably, uniform reconstruction techniques in the sub-muscular plane were employed for all patients by a seasoned surgeon, ensuring consistency between patients. Postmastectomy RT is increasingly indicated in patients with node-positive breast cancer. Several studies have reported that RT influences the quality and the results of the reconstructed breast, particularly with tissue expander techniques [12, 13]. In the present series, there was no correlation between RT and SCA deformity (Fig. 5). The median SCA change was found to be 2.05 in RT-treated breasts, while it was found to be 2.7 in RT-untreated breasts. These data are important because it includes high amount of radiotherapy patients compared to other sample studies in the literature. Based on the aforementioned data, we would like to state that radiotherapy has less impact in creating costal deformity compared to total expander volume. On the other hand, the fact that our RT positive and RT negative patient sample groups are not equal weakens this hypothesis. Preoperative preparation for breast reconstruction relies primarily on measuring anthropometrics and visually assessing the breasts. The absence of an objective evaluation of breast volume and shape could lead to less than optimal outcomes in reconstruction [14]. We believe that flattening of the costas also should be taken into consideration before second stage of surgery. There was no correlation between the size of the TE and the chest wall deformity in the present study. As can be seen from the graph (Fig. 6), no significant correlation was found. However, while the median angle change was 1.75 in 30 expanders inflated to ≤ 350 cc, the median angle change was 2.7 in expanders inflated to > 350 cc. Although this shows the increasing angle at high inflation amounts, it does not give a statistically significant value. The reason why a statistically significant result could not be reached may be that the expander inflation range of our patient group was narrow. It has been stated in the literature that costal deformities and even fractures were observed in publications where high-volume expander operations were performed. We attribute this deformity to the negative force created by the pectoral muscle and skin against expansion. This negative force can cause remodeling and even fracture in the ribs [15]. Considering the anatomical differences between the right and left sides of the ribcage, we compared the right- and left-sided SCA. While the median SCA change was calculated as 3.3 in the right breast, it was calculated as 1.95 in the left breast. We predicted that the thoracic deformity would be more prominent on the right breast expander applications due to the anatomical differences originating from the mediastinum; even though right breast showed more angle difference, we could not find a significant difference as a result of our analyses (Fig. 7). We observed that similar studies did not include this analysis and consider this evaluation as an important outcome of our study. We believe that prospective studies conducted in the light of these data will yield meaningful results in the future. Although we do not have enough patients to analyze the contribution of other accompanying chest soft tissue deformities to costal angulation, the importance of capsular contracture has been emphasized in similar studies in the literature [11, 16]. Our own study also demonstrates this situation in our examples of capsular contracture (Figs. 8 and 9). Focusing on this issue in future studies will be useful in understanding costal deformities.

This study has several limitations. Since the rib has a round and curved structure, determining the area of the rib where the angle measurement will be made may pose a problem in terms of the method. When deciding on the angle measurement area, we chose the area where the relevant rib showed the greatest slope in the anterior segment under the expander. This part of the measurement is operator dependent and may create limitations. The sample size was relatively small and homogeneous in terms of procedure. Larger samples with more heterogeneous samples, including older patients and different reconstructive protocols, may provide additional evidence of the effect of the use of TEs on chest wall morphology. We believe that additional studies with larger populations are warranted to understand the correlation between the tissue expander and chest wall deformities.

## Conclusion

The aim of this research was to investigate the relationship between the reconstructive use of a breast TE and chest wall abnormalities. Our findings provide additional evidence of a connection between the use of TEs and the emergence of chest wall deformities. Consequently, such deformities may arise sporadically, and there are no definitive associated conditions that can anticipate the chest wall's stability in breast reconstruction procedures involving a TE.
